# The Mediating Role of Resilience in the Association Between Social Interaction Anxiety and Self-Esteem Among Nursing Students in Clinical Settings

**DOI:** 10.3390/healthcare14172719

**Published:** 2026-08-26

**Authors:** Shaimaa Mohamed Amin, Mohamed Elsayed Ahmed Allawy, Radwa Ahmed Abdel Razek, Nagwa Yehya Ahmed Sabrah, Mohamed Hussein Ramadan Atta, Sally Mohammed Farghaly Abdelaliem, Nadiah A. Baghdadi, Eman Abdeen Ali, Mahmoud Abdelwahab Khedr

**Affiliations:** 1 Department of Community, Psychiatric, and Mental Health Nursing, College of Nursing, Qassim University, Buraydah 52571, Saudi Arabia; s.zaghlol@qu.edu.sa; 2 Community Health Nursing Department, Faculty of Nursing, Damanhour University, Damanhour 11222, Egypt; 3 Nursing Services Department, Qassim University Medical City, Buraydah 52571, Saudi Arabia; 4 Nursing Department, College of Applied Medical Sciences, Prince Sattam Bin Abdulaziz University, Wadi Addawasir 16273, Saudi Arabia; m.allawy@psau.edu.sa (M.E.A.A.); or mohamed-hussein@alexu.edu.eg (M.H.R.A.); 5 Psychiatric and Mental Health Nursing, RAK College of Nursing, RAK Medical and Health Sciences University, Ras Al Khaimah 11172, United Arab Emirates; radwa@rakmhsu.ac.ae; 6 Critical Care and Emergency Nursing Department, Faculty of Nursing, Mansoura University, Mansoura 35516, Egypt; nagwayehya@mans.edu.eg; 7 Psychiatric and Mental Health Nursing Department, Faculty of Nursing, Alexandria University, Alexandria 21526, Egypt; 8 Department of Nursing, Faculty of Allied Health Sciences, Kuwait University, Kuwait City 13060, Kuwait; sally.farghaly@alexu.edu.eg; 9 Department of Nursing Administration, Faculty of Nursing, Alexandria University, Alexandria 21526, Egypt; 10 Department of Nursing Management and Education, College of Nursing, Princess Nourah bint Abdulrahman University, P.O. Box 84428, Riyadh 11671, Saudi Arabia; nabaghdadi@pnu.edu.sa; 11 Medical-Surgical Nursing Department, Faculty of Nursing, Alexandria University, Alexandria 21526, Egypt; eman.abdeen@alexu.edu.eg

**Keywords:** students, social interaction anxiety, resilience, self-esteem, clinical practice

## Abstract

**Background**: Although social interaction anxiety, resilience, and self-esteem have each been investigated among nursing students, limited research has examined whether resilience contributes to the association between social interaction anxiety and self-esteem through an indirect pathway during clinical practice. Addressing this gap may improve the understanding of the psychological factors associated with students’ adaptation in clinical settings. Therefore, this study aimed to examine the relationships among social interaction anxiety, resilience, and self-esteem and the indirect role of resilience among nursing students. **Methods**: Using a cross-sectional descriptive research design, the study sampled 880 nursing students from two Egyptian universities through stratified random sampling. Participants completed the Connor–Davidson Resilience Scale, the Rosenberg Self-Esteem Scale, and the Social Interaction Anxiety Scale. An observed-score path analysis was used to examine the direct paths and the indirect pathway through resilience. **Results**: Social interaction anxiety was negatively correlated with self-esteem (r = −0.409, *p* < 0.001) and resilience (r = −0.134, *p* < 0.001), whereas resilience was positively correlated with self-esteem (r = 0.597, *p* < 0.001). In the observed-score path model, social interaction anxiety was negatively associated with resilience (B = −0.13, *p* < 0.001) and self-esteem (B = −0.18, *p* < 0.001), while resilience was positively associated with self-esteem (B = 0.32, *p* < 0.001). The product of the two component paths was small (approximately −0.04) and is reported descriptively as an indirect pathway. **Conclusions**: Resilience was positively associated with self-esteem and accounted for a small indirect component of the cross-sectional association between social interaction anxiety and self-esteem. This finding should not be interpreted as evidence of statistically significant or causal mediation.

## 1. Introduction 

Social anxiety disorder is recognized as one of the most common mental health disorders worldwide [1]. According to the *Diagnostic and Statistical Manual of Mental Disorders*, Fifth Edition (DSM-5), it is characterized by persistent fear or anxiety in social situations in which individuals may be exposed to scrutiny by others. This fear commonly stems from concerns about behaving in an embarrassing or humiliating manner or displaying visible symptoms of anxiety [2]. The prevalence of social anxiety varies across cultural settings, with reported rates ranging from 7% to 13% [3]. Anxiety is also common among university students, with evidence suggesting that more than one-third experience anxiety disorders [4].

Nursing students may be particularly vulnerable to social anxiety because their education requires frequent interpersonal interaction and continuous evaluation in both academic and clinical settings. Jia and Yue (2025) reported an average level of social anxiety among undergraduate nursing students, particularly females, with nearly half of the students experiencing symptoms ranging from mild to higher levels [5]. Such anxiety may interfere with academic performance and, importantly, with students’ ability to communicate confidently during clinical training.

Effective nursing practice depends heavily on communication, confidence, teamwork, and the ability to establish therapeutic relationships with patients and their families. Nursing students are expected to interact not only with patients but also with clinical instructors, staff nurses, physicians, and other members of the healthcare team. Therefore, social interaction anxiety may have consequences that extend beyond personal discomfort. It can interfere with students’ participation in clinical activities, willingness to ask questions, communication with healthcare professionals, and confidence in delivering patient care [6,7,8].

The clinical learning environment may intensify these difficulties because students are required to perform nursing procedures, communicate with unfamiliar patients, respond to questions, and demonstrate their competencies while being observed and evaluated. Clinical placements have consistently been described as one of the most stressful aspects of undergraduate nursing education [9,10]. Amsrud, Lyberg, and Severinsson (2019) identified academic, clinical, and personal sources of stress among final-year nursing students [11], while other studies have shown that assuming responsibility for patient care can evoke anxiety, worry, uncertainty, and fear of making mistakes [12,13]. More recent evidence also suggests that stress experienced within clinical learning environments may negatively influence students’ confidence, perceived competence, and clinical performance [14].

These demands make self-esteem particularly relevant to nursing education. Self-esteem reflects an individual’s overall evaluation of personal worth and competence and is influenced by how individuals perceive themselves in relation to their expectations and experiences [15]. For nursing students, self-esteem is not limited to general psychological well-being; it may also influence how they respond to feedback, interact with patients, approach clinical responsibilities, and perceive their own professional competence [7,16].

Previous studies have demonstrated meaningful associations between self-esteem and several educational and psychological outcomes among nursing students. The demands of nursing education, particularly interactions with faculty members and experiences of evaluation, may challenge students’ confidence and psychological well-being [17]. Almansour (2023) found that most nursing students had moderate self-esteem, although 15.3% were classified as having low self-esteem [18]. Higher self-esteem has also been associated with more effective stress management, resilience, and greater confidence in clinical situations [19,20].

Social interaction anxiety and self-esteem are closely related because students who are excessively concerned about being judged or evaluated may increasingly question their own abilities and personal worth. Previous evidence indicates that social anxiety is associated with negative self-perceptions and diminished self-worth [21,22]. Among nursing students specifically, lower levels of social anxiety have been associated with higher self-esteem [23]. Jia and Yue (2025) additionally found that social anxiety was positively associated with fears of both positive and negative evaluation and negatively associated with self-efficacy [5]. These findings suggest that the relationship between anxiety and self-esteem may involve broader psychological resources that shape how students respond to challenging interpersonal experiences.

One such resource is resilience. Resilience has received increasing attention in nursing education because nursing students encounter repeated academic, emotional, interpersonal, and clinical challenges throughout their training [10,24]. Rather than simply referring to the absence of stress, resilience describes the capacity to adapt positively, recover from difficulties, and maintain psychological functioning when confronted with adversity. Contemporary nursing literature increasingly views resilience as a dynamic and multidimensional process shaped by individual characteristics, coping strategies, social relationships, and educational experiences [25]. 

This perspective is particularly relevant during clinical practice. Nursing students do not encounter stress in isolation; they must continue communicating, learning, making decisions, and caring for patients while managing their own emotional responses. Resilience may therefore help students remain engaged in interpersonal and clinical situations despite anxiety, criticism, uncertainty, or perceived failure. A recent concept analysis of resilience among nursing students identified self-confidence, self-efficacy, coping with academic demands, adaptation to clinical practice, and use of social support as central attributes of resilience [26]. 

Earlier studies also support the importance of resilience in nursing education. Clinical experiences that generate anxiety, worry, and anger may influence students’ academic experiences and potentially their later professional development [13]. Student well-being has also been linked to professional competence and patient safety [12]. Although stress has traditionally been viewed mainly as harmful, manageable challenges accompanied by effective coping may contribute to adaptation and the development of psychological resources [12,27]. Delgado et al. (2017) emphasized the importance of resilience in helping nurses manage the emotional demands associated with professional practice [27], while Thomas and Revell (2016) argued that resilience development should be incorporated into nursing education to prepare students for sustainable careers [10]. Nevertheless, resilience-building strategies among nursing students remain an area requiring further investigation [24].

### 1.1. Knowledge Gap and Theoretical Basis

Although social interaction anxiety, self-esteem, and resilience have each been studied among nursing students, they have often been examined separately. This leaves an important question unanswered: why might students experiencing similar levels of social interaction anxiety differ in the extent to which their self-esteem is affected? Resilience may provide one explanation. The present study draws primarily on Conservation of Resources (COR) theory, which offers a more integrated explanation of the proposed relationships among the three variables [28]. COR theory proposes that individuals seek to obtain, maintain, and protect valued personal, psychological, and social resources. Stress is more likely to occur when these resources are threatened, lost, or perceived as insufficient to meet environmental demands. Applied to nursing students, the clinical environment places considerable demands on psychological resources. Students must communicate with unfamiliar people, demonstrate clinical competence, tolerate observation and evaluation, respond to criticism, and manage the possibility of making mistakes. For students with high social interaction anxiety, these repeated situations may consume emotional resources and reinforce negative perceptions of their competence. Over time, this pattern may be associated with poorer self-evaluation and lower self-esteem.

Within this framework, resilience can be understood as a protective personal resource that may help students adapt to these demands. A resilient student may still experience social interaction anxiety, but may be better able to recover from uncomfortable encounters, reinterpret negative experiences, tolerate evaluation, and continue participating in clinical activities. In contrast, students with fewer adaptive resources may experience greater difficulty recovering from socially threatening situations, potentially making the relationship between social interaction anxiety and negative self-evaluation stronger [26,28].

This theoretical position explains why resilience was examined as a potential indirect pathway rather than simply as another correlate. Social interaction anxiety may be associated with lower resilience because repeated fear of evaluation, avoidance, and interpersonal distress can interfere with adaptive engagement. Resilience, in turn, may be associated with stronger self-esteem because the ability to manage difficulties and recover from setbacks can reinforce feelings of competence and personal worth. Thus, resilience may account for part of the observed association between social interaction anxiety and self-esteem [29].

Existing empirical findings support this reasoning. Murad (2020) reported that individuals experiencing social anxiety commonly demonstrate negative self-perceptions and reduced self-worth [21], whereas Obadeji and Kumolalo (2022) emphasized the contribution of low self-esteem to social anxiety [30]. Among nursing students, social anxiety has been negatively associated with self-esteem [23], while greater social interaction anxiety has also been linked with poorer academic adjustment [31]. Resilience, conversely, has been associated with lower perceived stress and better psychological well-being among nursing students [24,32]. Resilience-focused educational interventions have also shown potential for strengthening students’ psychological adaptation [29]. 

The relationship may be especially important during clinical practice, where social interaction is unavoidable and directly connected to professional learning. Nursing students must approach patients, communicate sensitive information, work within multidisciplinary teams, seek clarification from supervisors, and perform skills in front of others. Fear of negative evaluation may therefore influence not only students’ emotional well-being but also their willingness to participate fully in learning opportunities. Previous research has shown that anxiety and fear during clinical practicums may influence the development of professional identity and that supportive relationships with preceptors and healthcare teams are important for students’ adjustment [33].

Recent Egyptian research has demonstrated that anxiety remains closely connected with educational and psychological characteristics among undergraduate nursing students [34]. Evidence from Egyptian nursing students also suggests that resilience is a potentially modifiable psychological resource rather than a fixed personal characteristic [24]. Despite these findings, limited evidence has examined social interaction anxiety, resilience, and self-esteem together within a single explanatory model among Egyptian nursing students during clinical practice. Previous studies have mainly investigated direct relationships between social anxiety and self-esteem [23], social anxiety and academic adjustment [31], or resilience and psychological well-being [25,32]. As a result, it remains unclear whether resilience statistically contributes to explaining the association between social interaction anxiety and self-esteem.

Examining this relationship may have practical value for nursing education. If resilience accounts for part of the association between social interaction anxiety and self-esteem, educational interventions may need to move beyond attempts to reduce anxiety alone. Supporting students’ adaptive capacities, confidence in handling interpersonal difficulties, and ability to recover from challenging clinical experiences may represent an additional approach to protecting psychological well-being and supporting professional development.

Accordingly, the present study aimed to examine the relationships among social interaction anxiety, resilience, and self-esteem among Egyptian nursing students during clinical practice and to examine whether resilience contributed to a small indirect association between social interaction anxiety and self-esteem.

### 1.2. Study Hypotheses

Based on the theoretical framework and previous evidence, the following hypotheses were proposed:

**H1:**  *Social interaction anxiety is negatively associated with self-esteem among nursing students*.

**H2:**  *Social interaction anxiety is negatively associated with resilience among nursing students*.

**H3:**  *Resilience is positively associated with self-esteem among nursing students*.

**H4:**  *The association between social interaction anxiety and self-esteem includes an indirect pathway through resilience, such that higher social interaction anxiety is associated with lower resilience, which in turn is associated with lower self-esteem*.

## 2. Methods

### 2.1. Design and Setting 

This study employed a cross-sectional descriptive design, following the guidelines outlined in the Strengthening the Reporting of Observational Studies in Epidemiology (STROBE) framework.

The study was conducted at the Faculties of Nursing of Zagazig University and Mansoura University, Egypt. Both faculties operate under the supervision of the Egyptian Ministry of Higher Education and adhere to national standards for nursing education. 

### 2.2. Participants 

Participants eligible for this study were nursing students enrolled at Zagazig University and Mansoura University in Egypt, who were currently engaged in clinical practice. Only those students who were willing to participate and provided informed consent were included in the study. Participants were required to have completed at least one clinical rotation to ensure that they had relevant experience for discussing their anxiety, resilience, and self-esteem in clinical settings. Students who were not actively participating in clinical practice during the study period were excluded, as they would not be able to provide relevant insights related to clinical anxiety and resilience. Additionally, Students with self-reported diagnosed psychological disorders or serious medical conditions that could influence social interaction anxiety, resilience, or self-esteem were excluded from the study.

#### Sample Size Calculation 

A stratified sampling technique was used to recruit nursing students and ensure representation across all academic years. The minimum required sample size was calculated using OpenEpi (version 3.01) based on a total population of 1000 students, a 99% confidence level, and a 5% margin of error, resulting in a minimum sample size of 622 participants [35]. To enhance representativeness and statistical precision, a total of 880 nursing students were ultimately included. The final sample exceeded the calculated minimum and was considered adequate for the reported regression and observed-score path analyses.

A stratified random sampling approach was used to select participants. First, the study population was divided by university and then by academic year to ensure that both universities and all four academic levels were represented. An equal number of students (440) was recruited from each university to provide comparable representation from the two study sites. Within each university, students were further grouped by academic year, and participants were selected systematically from the official student enrollment lists. For each stratum, the sampling interval was determined from the number of eligible students and the required stratum sample. Detailed stratum-specific eligible counts and sampling intervals were not available in the current reporting records and are therefore acknowledged as a limitation of sampling documentation.

A total of 900 eligible students were invited to participate. After data collection, 20 questionnaires were excluded because they were incomplete, leaving a final sample of 880 participants. This resulted in an overall response rate of 97.8%.

### 2.3. Study Measurements 

#### 2.3.1. The Demographic Form 

The questionnaire gathers socio-demographic details such as age, gender, academic year, place of residence, family structure, income level, and parents’ educational background.

#### 2.3.2. Connor–Davidson Resilience Scale (10-Item CD-RISC)

The Connor–Davidson Resilience Scale (CD-RISC), initially developed by Connor and Davidson in 2003, assesses resilience, or the ability to adapt and recover from adversity [36]. Campbell-Sills and Stein subsequently developed the 10-item version used in this study [37]. Each item is rated on a five-point Likert scale, ranging from “Not true at all” (1) to “True nearly all the time” (5), with higher scores indicating greater resilience. In the current study, the CD-RISC-10 showed strong internal consistency (Cronbach’s alpha = 0.905).

#### 2.3.3. Rosenberg Self-Esteem Scale

The Rosenberg Self-Esteem Scale (RSES), developed by Morris Rosenberg in 1965, is a widely used measure of global self-esteem [38]. It consists of 10 items, including five positively worded and five negatively worded statements. Each item is rated on a four-point Likert scale, ranging from “Strongly agree” (4) to “Strongly disagree” (1), with higher total scores indicating greater self-esteem after appropriate reverse scoring. In the current study, Cronbach’s alpha was 0.757.

#### 2.3.4. The Social Interaction Anxiety Scale (SIAS)

The Social Interaction Anxiety Scale (SIAS), developed by Mattick and Clarke in 1998, is a self-report questionnaire designed to assess fear and discomfort during social interactions [39]. The scale includes 20 items covering situations such as meeting new people, talking in groups, or initiating conversations. Each item is scored on a five-point Likert scale, ranging from “Not at all” (0) to “Extremely” (4), with higher total scores indicating greater social interaction anxiety. Items 5, 9, and 11 are reverse-scored. According to Šipka et al. (2023, the SIAS exhibited strong psychometric properties, including high internal consistency, with Cronbach’s alphas typically ranging from 0.85 to 0.92 [40]. In the current study, Cronbach’s alpha was 0.886.

### 2.4. Study Procedures

#### 2.4.1. Pilot Study

A pilot study involving 80 nursing students was conducted to assess the clarity, relevance, and reliability of the translated Arabic instruments; these participants were excluded from the main study. 

#### 2.4.2. Data Collection 

Data collection took place from January to March 2025. The process commenced with an orientation session led by the researcher, during which the study’s purpose was clearly outlined, and participants were informed that their involvement was entirely voluntary. All inquiries or concerns were thoroughly addressed, and assurances of confidentiality were emphasized to foster participants’ trust. Data were collected through structured, self-administered questionnaires, distributed in various campus locations such as lecture halls and libraries, from Sunday to Thursday. Both verbal and written informed consent were obtained prior to participation. On average, completing the questionnaire required 10 to 15 min.

### 2.5. Statistical Analysis 

IBM SPSS 26 and AMOS 23 (IBM Corp., Armonk, NY, USA) were used for analysis. Descriptive statistics were in terms of means, standard deviations, frequencies, and percentages. Pearson correlations were computed for social interaction anxiety, self-esteem, and resilience. A Hierarchical multiple regression analysis predicted self-esteem. Three contextual factors (father’s educational level, number of clinical rotations, and urban/rural residence) were reported as predictors of the model. Then, the variables of social interaction anxiety and resilience were added sequentially. The demographic measures that were not measured were not entered and are considered potential sources of residual confounding. For clinical rotation, the variable was consistently coded as one rotation versus two or more rotations for the regression analysis. The multi-collinearity was assessed using the variance inflation factor (VIF), and the Durbin-Watson statistic (DW) was reported to assess the residual independence. The AMOS analysis was an observed-score path model with no covariates and four composite scale scores in the three-variable path model. Global fit indices (CFI, TLI, RMSEA, SRMR, and chi-square/df) are not informative as the path model was saturated (df = 0). The indirect pathway was reported descriptively as the product of the two component paths (a × b) but not bootstrapped and no confidence interval computed. For this reason, the product term should not be taken as evidence of a difference between the indirect effect and 0. Therefore, the product term should not be interpreted as an indication of a difference between the indirect effect and 0. All analyses reported use the entire sample (*n* = 880). For the individual regression and path coefficients, a *p*-value < 0.05 (two-tailed) was regarded as statistically significant.

### 2.6. Ethical Considerations and Consent to Participate 

The research received ethical clearance from the Research Ethics Committee at the Faculty of Nursing, Zagazig University, Egypt, under approval number 239. Administrative permission to collect data at the Faculty of Nursing, Mansoura University was also obtained from the relevant university authority. Thus, the manuscript distinguishes the study’s ethical clearance from the administrative authorization for the second data-collection site. Consistent with the Declaration of Helsinki, all participants were informed about the study purpose and procedures. Voluntary participation, confidentiality, anonymity, and the right to withdraw without adverse consequences were maintained throughout the study.

## 3. Results

Table 1 indicates that females comprised 55.8% of the student population, while males accounted for 44.2%. The average age was 18.94 years (SD = 1.77). The majority of students were single, comprising 95.9%, while a smaller fraction were married at 3.4%, and a minimal 0.7% were widowed. In terms of residence, 66.9% were located in rural areas, while 33.1% resided in urban areas. A total of 42.6% of the mothers and 36.1% of the fathers of the students had attained secondary education. Furthermore, 28.7% of mothers and 29.9% of fathers achieved university education or higher. A lesser percentage of mothers (19.7%) and fathers (24.5%) possessed basic education, whereas 9.0% of mothers and 9.4% of fathers were found to be illiterate. A majority of students were part of extended families, accounting for 58.2%, while 41.8% were from nuclear families. Regarding family income, 78.3% indicated they have sufficient income, whereas 21.7% reported that it is inadequate. A breakdown of clinical experience reveals that 40.0% of the students completed one clinical rotation, 21.0% completed three, 18.8% completed two, 8.8% completed four, and 11.5% completed five or more rotations.

**Figure 1:**
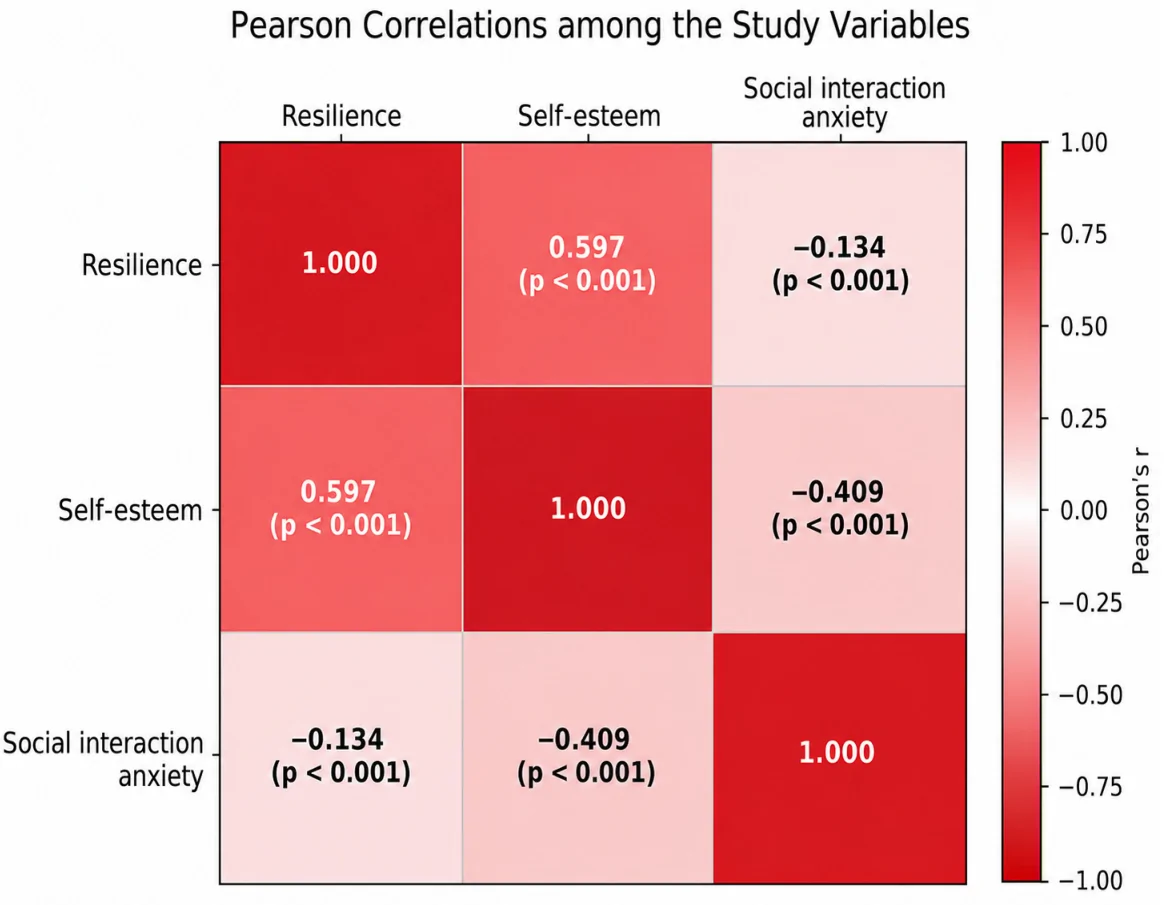
Pearson correlation matrix among resilience, self-esteem, and social interaction anxiety (*n* = 880).

Figure 1 presents the Pearson correlation matrix for resilience, self-esteem, and social interaction anxiety (*n* = 880). Resilience was positively correlated with self-esteem (r = 0.597, *p* < 0.001) and negatively correlated with social interaction anxiety (r = −0.134, *p* < 0.001). Self-esteem was negatively correlated with social interaction anxiety (r = −0.409, *p* < 0.001).

**Figure 2:**
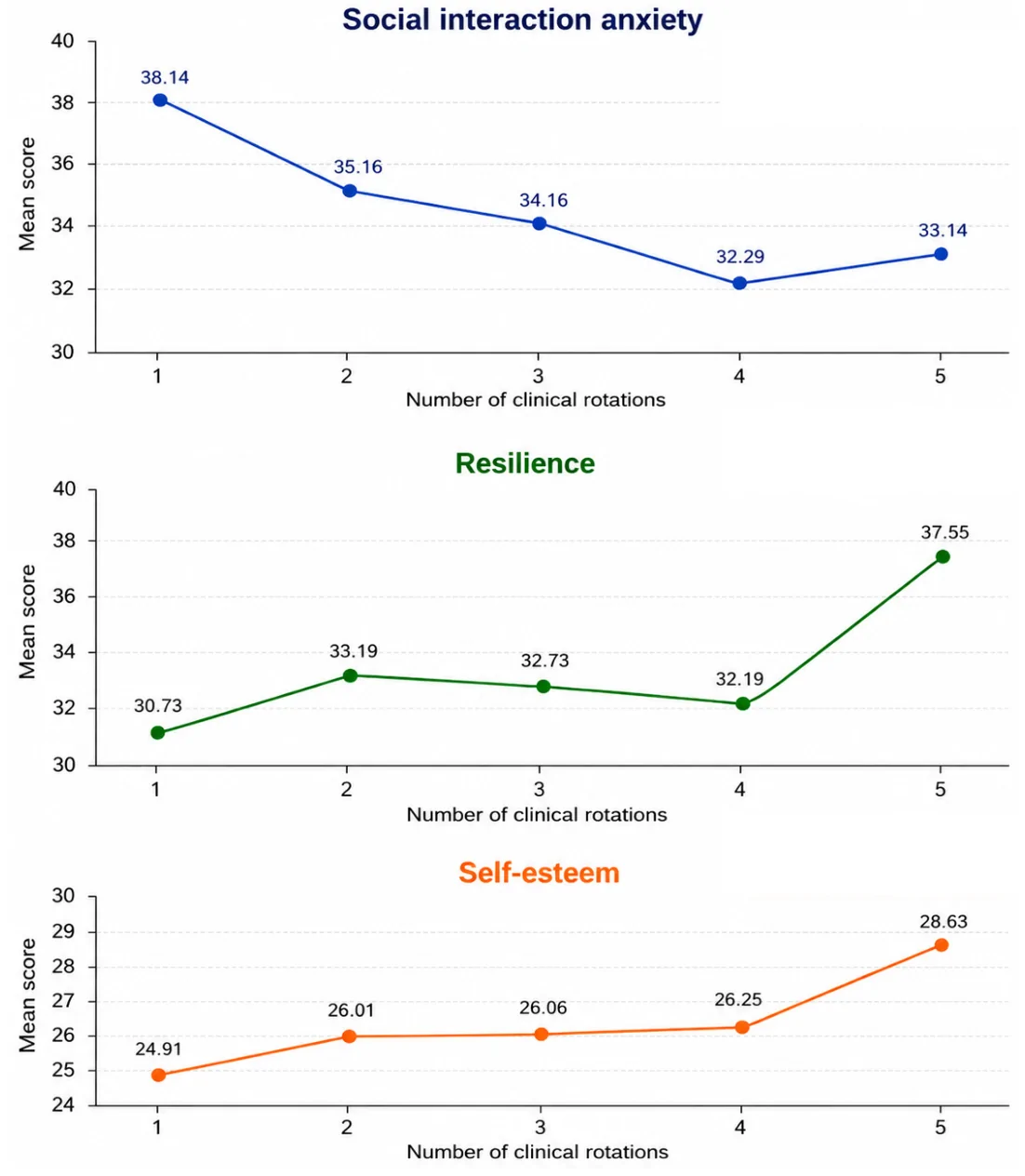
Descriptive Mean Scores of Social Interaction Anxiety, Resilience, and Self-Esteem across Clinical-Rotation Groups (*n* = 880).

Figure 2 presents descriptive mean scores across the independent clinical-rotation groups. Social interaction anxiety decreased from 38.14 among students with one rotation to 32.29 among those with four rotations, with a slight increase to 33.14 among those with five or more rotations. Resilience varied from 30.73 in the one-rotation group to 37.55 in the five-or-more-rotations group, while self-esteem ranged from 24.91 to 28.63 across these groups. These values are presented descriptively only; no repeated-measures analysis or inferential between-group comparison is reported for Figure 2.

Table 2 and Table 3 present the hierarchical multiple regression results. Model 1, which included father’s educational level (university or above vs. lower educational levels), explained 1.4% of the variance in self-esteem (R^2^ = 0.014; B = 1.59, *p* = 0.001). Adding the clinical-rotation category (one rotation vs. two or more) in Model 2 increased R^2^ to 0.028 (R^2^ change = 0.014; B = −1.48, *p* = 0.001). Adding residence in Model 3 increased R^2^ to 0.035 (R^2^ change = 0.007), with urban residence associated with a higher self-esteem score (B = 1.06, *p* = 0.019).

**Figure 3:**
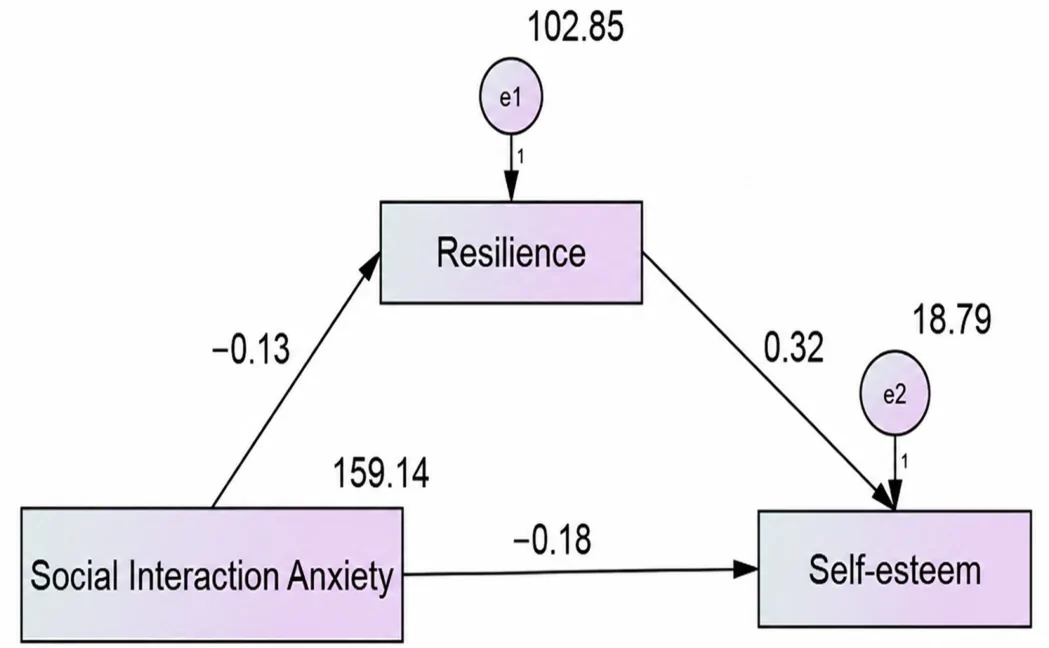
Observed-score path analysis for social interaction anxiety, resilience, and self-esteem (*n* = 880). Values on arrows are unstandardized coefficients (B). The model is saturated (df = 0); therefore, global fit indices are not informative. The indirect pathway is reported descriptively and was not tested with bootstrap confidence intervals.

Adding social interaction anxiety in Model 4 increased the explained variance by 19.2% (R^2^ change = 0.192), and social interaction anxiety was negatively associated with self-esteem (B = −0.22, β = −0.446, *p* < 0.001). In Model 5, social interaction anxiety remained negatively associated with self-esteem (B = −0.19, β = −0.382, *p* < 0.001), whereas resilience was positively associated with self-esteem (B = 0.33, β = 0.543, *p* < 0.001). The final model explained 50.6% of the variance in self-esteem (adjusted R^2^ = 0.503). Father’s educational level, clinical-rotation category, and residence were not statistically significant in the final model. The Durbin-Watson statistic was 1.828 and VIF values ranged from 1.0 to 1.3. Figure 3 shows the observed-score path analysis with unstandardized coefficients (B). Social interaction anxiety was negatively associated with resilience (B = −0.13, *p* < 0.001) and self-esteem (B = −0.18, *p* < 0.001), while resilience was positively associated with self-esteem (B = 0.32, *p* < 0.001). The product of the two component paths was small (approximately −0.04) and is reported only as a descriptive indirect pathway because no bootstrap confidence interval for the indirect effect was calculated.

## 4. Discussion

Nursing students encounter multiple academic, interpersonal, and clinical demands that may be associated with social interaction anxiety and self-esteem. This study examined the relationships among social interaction anxiety, resilience, and self-esteem among Egyptian nursing students during clinical practice and explored whether resilience contributed to a small indirect association between social interaction anxiety and self-esteem.

The correlation pattern indicates that resilience was associated with more positive self-evaluation and lower social interaction anxiety among nursing students. One possible explanation is that students with greater resilience may be better able to adapt to academic and clinical pressures, recover from difficult experiences, and maintain psychological functioning. These findings can be interpreted within Maslow’s hierarchy of needs, which identifies self-esteem as an important psychological need related to personal growth and self-actualization [22]. However, because the present study is cross-sectional, these associations should not be interpreted as evidence that resilience causes improvements in self-esteem or reduces social interaction anxiety.

Previous research has similarly identified resilience as an important psychological resource associated with nursing students’ responses to clinical and academic demands [24,32]. Evidence that higher social anxiety is associated with lower self-esteem among undergraduate nursing students is also consistent with the inverse relationship observed in this study [23]. Interpersonal challenges and fear of negative evaluation may be one possible explanation for this association. The negative association between social interaction anxiety and resilience likewise suggests that students reporting stronger adaptive resources tended to report less anxiety in socially demanding situations; however, the direction of influence cannot be determined from the present design.

Descriptively, the clinical-rotation groups showed a pattern of lower mean social interaction anxiety and higher mean resilience and self-esteem in some groups with greater clinical exposure. Repeated clinical experiences may provide opportunities to become more familiar with clinical settings, communication demands, and coping strategies [41,42]; however, the present descriptive comparison does not establish that clinical exposure itself produced these differences. The variation across intermediate groups also indicates that the pattern was not uniformly linear. Future longitudinal or appropriately tested between-group studies are needed to determine whether these psychological outcomes change with increasing clinical exposure.

The hierarchical regression findings indicate that self-esteem among nursing students was more strongly related to psychological factors than to background or educational characteristics. Although paternal educational level, number of clinical rotations, and place of residence initially contributed modestly to the explanation of self-esteem, their effects were substantially attenuated and became non-significant after social interaction anxiety and resilience were entered into the model. This pattern implies that rather than demographic traits alone, students’ self-esteem may be more strongly linked to their present psychological resources and interpersonal experiences. Specifically, the negative correlation between social interaction anxiety and self-esteem is in line with earlier research among undergraduate nursing students that indicates higher levels of social anxiety are linked to lower levels of self-esteem, possibly because positive self-appraisal is undermined by interpersonal challenges and fear of negative evaluation [21,23]. Conversely, resilience emerged as the strongest predictor in the final model, supporting evidence that resilience represents an important psychological resource for nursing students and is linked to better psychological functioning and adaptation to academic and clinical stressors [24,27]. In the present cross-sectional analysis, however, “predictor” refers to its statistical contribution to the regression model and should not be interpreted as indicating that resilience causes higher self-esteem. The marked improvement in the explanatory power of the model after adding these psychological variables further emphasizes their relevance to self-esteem, but this increase in explained variance does not establish temporal or causal relationships among the variables.

The observed-score path analysis further illustrates the interconnectedness of social interaction anxiety, resilience, and self-esteem among nursing students. Social interaction anxiety was negatively associated with resilience and self-esteem, whereas resilience was positively associated with self-esteem. These directions are consistent with previous evidence concerning social anxiety, resilience, and self-appraisal among nursing students [23,24,29,32,43]. The product of the social interaction anxiety-to-resilience and resilience-to-self-esteem paths was small, suggesting a possible indirect pathway through resilience. This finding is descriptive only and should not be interpreted as statistically significant or causal mediation because the study was cross-sectional and no bootstrap confidence interval for the indirect effect was calculated.

## 5. Strengths and Limitations

This study has several strengths. First, the relatively large sample of 880 nursing students increased the precision of the reported estimates. Second, stratified sampling allowed students from different academic years to be represented. Third, recruiting participants from two nursing faculties provided evidence from more than one educational setting. Fourth, the study used established instruments that demonstrated acceptable-to-strong internal consistency in the current sample.

Several limitations should be considered when interpreting the findings. The cross-sectional design does not allow temporal or causal relationships to be established among social interaction anxiety, resilience, and self-esteem; therefore, the observed indirect pathway should not be interpreted as evidence of statistically significant or causal mediation. No bootstrap confidence interval was calculated for the indirect effect. The use of self-report questionnaires may have introduced reporting or social desirability bias. Students with self-reported psychological disorders or serious medical conditions were excluded, which may limit generalizability to students experiencing such conditions. Although participants were recruited from two universities, the study involved only nursing students in Egypt. Other unmeasured factors, including personality, social support, academic stress, and previous psychological experiences, may also have contributed to the observed associations. Future longitudinal, multi-center, and intervention-based studies are recommended to clarify temporal relationships, validate the measurement structure in comparable Arabic-speaking samples, and test whether targeted interventions influence resilience, social interaction anxiety, or self-esteem.

## 6. Conclusions

This study found that social interaction anxiety, resilience, and self-esteem were closely related among nursing students during clinical practice. Students who reported greater social interaction anxiety tended to report lower self-esteem and resilience, while those with greater resilience tended to report higher self-esteem. Resilience also formed part of a small descriptive indirect pathway between social interaction anxiety and self-esteem. Because the study was cross-sectional and the indirect effect was not evaluated with a bootstrap confidence interval, this pathway should not be interpreted as evidence of statistically significant or causal mediation. The findings support further longitudinal and intervention research on resilience, social interaction anxiety, and self-esteem in nursing education.

## 7. Relevance for Clinical Practice

The findings suggest that resilience and social interaction anxiety are relevant considerations in nursing education and clinical learning environments. Nursing educators may consider incorporating opportunities for students to develop adaptive coping, communication, and interpersonal skills through activities such as simulation, role-play, and guided reflection. Supportive relationships with clinical instructors and peers may also provide students with a more comfortable environment in which to develop confidence and engage in clinical interactions. In clinical settings, creating a supportive and psychologically safe learning environment may help students participate more comfortably and seek guidance when they experience difficulties in social or clinical situations. 

Clinical instructors and preceptors may also benefit from being aware of students who experience difficulties with social interactions and from using supportive and constructive approaches during clinical supervision. However, these implications should be considered as potential educational directions rather than established intervention effects, as the present cross-sectional study cannot determine whether resilience-building or communication-focused interventions would reduce social interaction anxiety or increase self-esteem. Future longitudinal and intervention-based studies are needed to evaluate the effectiveness of such approaches and to examine how resilience, social interaction anxiety, and self-esteem change throughout nursing students’ academic and clinical experiences.

Ethical Considerations and consent to participate: The research received ethical clearance from the Research Ethics Committee at the Faculty of Nursing, Zagazig University, Egypt, under approval number 239. Administrative permission for data collection at the Faculty of Nursing, Mansoura University was obtained from the relevant university authority. All participants were informed about the study purpose and procedures, and voluntary participation, confidentiality, anonymity, and the right to withdraw were maintained in accordance with the Declaration of Helsinki.

## Figures and Tables

**Table 1:** Distribution of the nursing students regarding their demographic characteristics (*n* = 880).

Variables	Categories	N (%)
GENDER	Male	389 (44.2)
	Female	491 (55.8)
AGE, YEARS MEAN ± SD	Mean ± SD	18.94 ± 1.77
MARITAL STATUS	Single	844 (95.9)
	Married	30 (3.4)
	Widowed	6 (0.7)
RESIDENCE	Urban	291 (33.1)
	Rural	589 (66.9)
MOTHER’S EDUCATION	Illiterate	79 (9.0)
	Basic	173 (19.7)
	Secondary	375 (42.6)
	University or above	253 (28.7)
FATHER’S EDUCATION	Illiterate	83 (9.4)
	Basic	216 (24.5)
	Secondary	318 (36.1)
	University or above	263 (29.9)
FAMILY TYPE	Nuclear	368 (41.8)
	Extended	512 (58.2)
FAMILY INCOME	Enough	689 (78.3)
	Not enough	191 (21.7)
CLINICAL ROTATIONS COMPLETED	1	352 (40.0)
	2	165 (18.8)
	3	185 (21.0)
	4	77 (8.8)
	5 OR MORE	101 (11.5)

**Table 2:** Model summary for the hierarchical multiple regression predicting self-esteem among nursing students (*n* = 880).

Model	R	R^2^	Adjusted R^2^	Std. Error of the Estimate	Change Statistics			Durbin-Watson	VIF
					ΔR^2^	F Change	Sig. F Change		
1	0.119 ^a^	0.014	0.013	6.08	0.014	11.82	0.001	1.828	1.0
2	0.168 ^b^	0.028	0.026	6.04	0.014	11.94	0.001		1.0
3	0.186 ^c^	0.035	0.031	6.02	0.007	5.56	0.019		1.1
4	0.476 ^d^	0.226	0.223	5.39	0.192	203.41	<0.001		1.2
5	0.712 ^e^	0.506	0.503	4.31	0.280	465.36	<0.001		1.3

^a^. Predictors: (Constant), Father’s Educational Level. ^b^. Predictors: (Constant), Father’s Educational Level, Number of Clinical Rotations Completed. ^c^. Predictors: (Constant), Father’s Educational Level, Number of Clinical Rotations Completed, Place of Residence. ^d^. Predictors: (Constant), Father’s Educational Level, Number of Clinical Rotations Completed, Place of Residence, Social interaction anxiety. ^e^. Predictors: (Constant), Father’s Educational Level, Number of Clinical Rotations Completed, Place of Residence, Social interaction anxiety, Resilience.

**Table 3:** Multiple regression coefficients for the predictors of self-esteem among nursing students (*n* = 880).

Model		Unstandardized Coefficients		Standardized Coefficients	t	*p*-Value	95.0% Confidence Interval for B	
		B	Std. Error	β			Lower Bound	Upper Bound
1	(Constant)	25.43	0.25		100.69	<0.001 *	24.93	25.92
	Father’s Educational Level (high)	1.59	0.46	0.119	3.44	0.001	0.68	2.49
2	(Constant)	26.02	0.30		85.85	<0.001 *	25.42	26.61
	Father’s Educational Level (high)	1.63	0.46	0.122	3.55	<0.001 *	0.73	2.53
	Clinical rotations completed (1 vs. ≥2)	−1.48	0.43	−0.119	−3.46	0.001	−2.33	−0.64
3	(Constant)	24.29	0.79		30.66	<0.001 *	22.73	25.84
	Father’s Educational Level (high)	1.65	0.46	0.123	3.60	<0.001 *	0.75	2.55
	Clinical rotations completed (1 vs. ≥2)	−1.595	0.43	−0.128	−3.71	<0.001 *	−2.44	−0.75
	Place of Residence (urban)	1.06	0.45	0.081	2.36	0.019	0.18	1.94
4	(Constant)	32.67	0.92		35.46	<0.001 *	30.86	34.48
	Father’s Educational Level (high)	1.14	0.41	0.085	2.76	0.006	0.33	1.95
	Clinical rotations completed (1 vs. ≥2)	−0.68	0.39	−0.054	−1.73	0.084	−1.44	0.09
	Place of Residence (urban)	0.55	0.40	0.042	1.36	0.175	−0.24	1.34
	Social Interaction Anxiety	−0.22	0.02	−0.446	−14.26	<0.001 *	−0.25	−0.19
5	(Constant)	21.41	0.90		23.71	0.001 *	19.63	23.18
	Father’s Educational Level (high)	0.358	0.33	0.027	1.08	0.280	−0.29	1.01
	Clinical rotations completed (1 vs. ≥2)	0.23	0.32	0.018	0.72	0.470	−0.39	0.84
	Place of Residence (urban)	0.198	0.32	0.015	0.61	0.539	−0.44	0.83
	Social Interaction Anxiety	−0.19	0.01	−0.382	−15.16	<0.001 *	−0.21	−0.16
	Resilience	0.33	0.02	0.543	21.57	<0.001	0.30	0.36

* significant at *p*-value < 0.05.

## Data Availability

The data presented in this study are available on request from the corresponding author due to privacy, confidentiality concerns, and ethical guidelines restricting data distribution.

## References

[1] Hajure M., Tariku M., Abdu Z. (2020). Prevalence and associated factors of social phobia among college of health science students, Mettu town, southwest Ethiopia, 2019; institutional-based cross-sectional study. Open Public Health J..

[2] APA (2013). Diagnostic and Statistical Manual of Mental Disorders.

[3] Alkhalifah A.K., Alsalameh N.S., Alhomaidhy M.A., Alrwies N.A. (2017). Prevalence of social phobia among medical students in Saudi Arabia. Egypt. J. Hosp. Med..

[4] Tan G.X., Soh X.C., Hartanto A., Goh A.Y., Majeed N.M. (2023). Prevalence of anxiety in college and university students: An umbrella review. J. Affect. Disord. Rep..

[5] Jia Y., Yue Y. (2025). Influencing Factors of Social Anxiety of Undergraduate Nursing Students Based on Random Forest Model: A Cross-Sectional Study. Nurs. Open.

[6] Toqan D., Ayed A., Amoudi M., Alhalaiqa F., Alfuqaha O.A., ALBashtawy M. (2022). Effect of progressive muscle relaxation exercise on anxiety among nursing students in pediatric clinical training. SAGE Open Nurs..

[7] Dancot J., Pétré B., Detroz P., Gagnayre R., Dardenne N., Donneau A.-F., Guillaume M. (2020). Exploring nursing student self-esteem and its relationship to clinical competence development: Protocol for a multiphase convergent mixed methods study. Int. J. Nurs. Clin. Pract..

[8] Ghezelbash S., Rahmani F., Peyrovi H., Inanloo M. (2014). Study of social anxiety in nursing students of Tehran University of Medical Sciences. Res. Dev. Med. Educ..

[9] Turner K., McCarthy V.L. (2017). Stress and anxiety among nursing students: A review of intervention strategies in the literature between 2009 and 2015. Nurse Educ. Pract..

[10] Thomas L.J., Revell S.H. (2016). Resilience in nursing students: An integrative review. Nurse Educ. Today.

[11] Amsrud K.E., Lyberg A., Severinsson E. (2019). Development of resilience in nursing students: A systematic qualitative review and thematic synthesis. Nurse Educ. Pract..

[12] Gibbons C., Dempster M., Moutray M. (2009). Index of sources of stress in nursing students: A confirmatory factor analysis. J. Adv. Nurs..

[13] Reeve K.L., Shumaker C.J., Yearwood E.L., Crowell N.A., Riley J.B. (2013). Perceived stress and social support in undergraduate nursing students’ educational experiences. Nurse Educ. Today.

[14] Welch S.R. (2023). Clinical stress and clinical performance in prelicensure nursing students: A systematic review. J. Nurs. Educ..

[15] Guindon M.H. (2009). Self-Esteem Across the Lifespan: Issues and Interventions.

[16] Rania N., Siri A., Bagnasco A., Aleo G., Sasso L. (2014). Academic climate, well-being, and academic performance in a university degree course. J. Nurs. Manag..

[17] Dimitriadou-Panteka A., Koukourikos K., Pizirtzidou E. (2014). The concept of self-esteem in nursing education and its impact on professional behavior. Int. J. Caring Sci..

[18] Almansour A.M. (2023). Self-esteem among nursing students at a public university in Saudi Arabia: A cross-sectional study. Belitung Nurs. J..

[19] Rosy O., Nandini M., Princy M., Divya N. (2022). Objective Structured Clinical Exam: A Cross-Sectional Study To Evaluate Undergraduate Nursing Students’ Experience. Int. J. Nurs. Care.

[20] Bibi A., Kahliq F., Younus M. (2024). Explore the level of self-esteem among undergraduate nursing students. J. Xi’an Shiyou Univ. Nat. Sci. Ed..

[21] Murad O.S. (2020). Social Anxiety in Relation to Self-Esteem among University Students in Jordan. Int. Educ. Stud..

[22] Taneva T. (2023). Revisions to Maslow’s hierarchical model of basic psychological needs. Trakia J. Sci..

[23] Ayed A., Abu Ejheisheh M., Batran A., Albashtawy M., Salameh W.A., Obeyat A.H., Melhem R.H., Shawawrha I.O., Batran A. (2024). Relationship between social anxiety and self-esteem among undergraduate nursing students. Inq. J. Health Care Organ. Provis. Financ..

[24] Khedr M.A., Alharbi T.A., Alkaram A.A., Hussein R.M. (2023). Impact of resilience-based intervention on emotional regulation, grit and life satisfaction among female Egyptian and Saudi nursing students: A randomized controlled trial. Nurse Educ. Pract..

[25] Aryuwat P., Asp M., Lövenmark A., Radabutr M., Holmgren J. (2023). An integrative review of resilience among nursing students in the context of nursing education. Nurs. Open.

[26] Park S., Choi M.Y. (2025). Resilience of nursing students: A concept analysis study. Nurse Educ. Today.

[27] Delgado C., Upton D., Ranse K., Furness T., Foster K. (2017). Nurses’ resilience and the emotional labor of nursing work: An integrative review of empirical literature. Int. J. Nurs. Stud..

[28] Hobfoll S.E., Halbesleben J., Neveu J.P., Westman M. (2018). Conservation of resources in the organizational context: The reality of resources and their consequences. Annu. Rev. Organ. Psychol. Organ. Behav..

[29] Ejaz H., Sultan B., Pienaar A.J., Froelicher E.S. (2024). Effectiveness of a resilience-focused educational program for promoting resilience in nursing students: A systematic review and meta-analysis. Nurse Educ. Pract..

[30] Obadeji A., Kumolalo B.F. (2022). Social anxiety disorder among undergraduate students: Exploring association with self-esteem and personality traits. World Soc. Psychiatry.

[31] Sharma N., Thakur M. (2022). Occurrence of social interaction anxiety and its association with academic adjustment. Int. J. Appl. Res..

[32] Li Z.-S., Hasson F. (2020). Resilience, stress, and psychological well-being in nursing students: A systematic review. Nurse Educ. Today.

[33] Araújo A., Godoy S., Ventura C., Silva I., Almeida E., Mendes I. (2022). Reflections on nursing students’ fear and anxiety arising from clinical practicums. Investig. Educ. Enferm..

[34] Ibrahim F.M., Elhabashy H.M. (2025). Relationship between academic performance, personality traits, and anxiety level among Egyptian undergraduate nursing students: A correlational research study. BMC Nurs..

[35] Sullivan K.M., Dean A., Soe M.M. (2009). On academics: OpenEpi: A web-based epidemiologic and statistical calculator for public health. Public Health Rep..

[36] Connor K.M., Davidson J.R. (2003). Development of a new resilience scale: The Connor-Davidson resilience scale (CD-RISC). Depress. Anxiety.

[37] Campbell-Sills L., Stein M.B. (2007). Psychometric analysis and refinement of the Connor–Davidson Resilience Scale (CD-RISC): Validation of a 10-item measure of resilience. J. Trauma. Stress Off. Publ. Int. Soc. Trauma. Stress Stud..

[38] Rosenberg M. (1965). Rosenberg self-esteem scale (RSE). Accept. Commit. Ther. Meas. Package.

[39] Mattick R.P., Clarke J.C. (1998). Development and validation of measures of social phobia, scrutiny fear, and social interaction anxiety. Behav. Res. Ther..

[40] Šipka D., Brodbeck J., Schulz A., Stolz T., Berger T. (2023). Factor structure of the Social Phobia Scale (SPS) and the Social Interaction Anxiety Scale (SIAS) in a clinical sample recruited from the community. BMC Psychiatry.

[41] Amin S.M., El-Ashry A.M., Karim N.A., Atta M.H., Alqarawi N., Khedr M.A., Abozed H.W., Ali E.A., Mohamed A.Z., El-Monshed A.H. (2026). Shaping competence through identity: The mediating role of professional identity in the relationship between clinical learning environment and nursing competence among nursing students. Nurse Educ. Today.

[42] Dias J.M., Subu M.A., Al-Yateem N., Ahmed F.R., Rahman S.A., Abraham M.S., Forootan S.M., Sarkhosh F.A., Javanbakh F. (2024). Nursing students’ stressors and coping strategies during their first clinical training: A qualitative study in the United Arab Emirates. BMC Nurs..

[43] Mendes P.R., de Araújo A.T., Bastos P.B., Neuhauss E., Monteiro L.Z., Rauber S.B. (2021). Perceived self-esteem, resilience and stress of students entering a nursing degree. Acta Sci. Health Sci..

